# Engineering an efficient and bright split *Corynactis californica* green fluorescent protein

**DOI:** 10.1038/s41598-021-98149-8

**Published:** 2021-09-16

**Authors:** Hau B. Nguyen, Thomas C. Terwilliger, Geoffrey S. Waldo

**Affiliations:** 1 Bioscience Division, MS M888, Los Alamos National Laboratory, Los Alamos, NM 87545 USA; 2 New Mexico Consortium, 100 Entrada Dr, Los Alamos, NM 87544 USA

**Keywords:** Biochemistry, Biotechnology, Molecular biology

## Abstract

Split green fluorescent protein (GFP) has been used in a panoply of cellular biology applications to study protein translocation, monitor protein solubility and aggregation, detect protein–protein interactions, enhance protein crystallization, and even map neuron contacts. Recent work shows the utility of split fluorescent proteins for large scale labeling of proteins in cells using CRISPR, but sets of efficient split fluorescent proteins that do not cross-react are needed for multiplexing experiments. We present a new monomeric split green fluorescent protein (ccGFP) engineered from a tetrameric GFP found in *Corynactis californica,* a bright red colonial anthozoan similar to sea anemones and scleractinian stony corals. Split ccGFP from *C. californica* complements up to threefold faster compared to the original *Aequorea victoria* split GFP and enable multiplexed labeling with existing *A. victoria* split YFP and CFP.

## Introduction

Full-length fluorescent proteins are widely used as fusion tags. The use of small epitope tags can reduce functional perturbation caused by bulky tags and enable signal amplification using labeled antibodies, but washing is needed to remove unbound labeled antibodies to reduce background. Small fragments of split fluorescent proteins^1^ can be used in lieu of epitope tags with the remaining large fragment acting as a ‘signaling antibody’, eliminating the need to wash away unbound labeled antibody and side-stepping the background signal problem associated with conventional labeled antibodies^2^. The first efficient, self-assembling two-part split GFP was developed^1^ from *Aequorea victoria* GFP (referred to as ‘GFP’) during the first phase of the NIH-funded Protein Structure Initiative to screen the expression and solubility of large numbers of different proteins in heterologous hosts such as *E. coli*. This system uses strand 11 (S11) from GFP as a fusion tag, and the remaining strands (GFP 1–10) as a detector. Extensive engineering yielded a GFP S11 tag with minimal effect on the proteins it was attached to, and a GFP 1–10 that remained soluble prior to becoming fluorescent only upon binding to S11. The system rapidly and spontaneously assembles with picomolar affinity (without the need for attached interacting proteins) to form the folded 11-stranded GFP beta barrel^1^, followed by the usual chromophore maturation (t_1/2_ ~ 10 min). We previously described a new system for studying molecular interactions that uses interacting domains to drive assembly of a three-part split GFP^3^. These split GFPs have been widely used in cellular biology to study neuron gap junction formation^4^, observe viral fusion with host cells^5^, label PB2 protein in replication competent influenza-A^6^, determine membrane protein topology and compartmentalization in plasmodia^7^, monitor pathogen effector protein trafficking in host cells^8,9^, detect protein-RNA interactions^10,11^, track macromolecule delivery into live cells^12^, label GFP S11-tagged proteins in cells using GFP 1–10 as a ‘signaling’ antibody^2^, study light-activated disassembly/assembly of GFP^13–15^, make efficient protease sensors^16^, test topological ‘rewiring’ of proteins^17^, create self-assembling nanostructures^18^, to efficiently label and detect human proteins using CRISPR/cas9^19^, and even to form artificial complexes for assisting protein crystallization^20^.

In order to make split fluorescent protein labeling of proteins in living cells even more widely applicable, several technical advances are needed, including new approaches to insert the split protein tags^19^, faster complementing split fluorescent proteins, and additional orthogonal split fluorescent proteins that do not ‘cross-react’ (i.e., the large detector fragment from one does not efficiently bind and fold with the small beta strand fragment from another) and that have spectrally-distinct colors. Along these lines, Bo Huang and co-workers applied the CRISPR-cas9 to label a plurality of human proteins using split GFP^19^ along with a split superfolder mCherry red fluorescent protein (RFP)^20^, and improved split Cherry RFP variants^21^, further highlighting the potential impact of additional split fluorescent proteins for multiplex labeling in living cells.

In this work, we demonstrate a new split fluorescent protein system engineered from *C. californica* GFP (referred to as ccGFP), optimized to minimize fusion protein perturbation by the ccGFP S11 tag and ensure that the ccGFP 1–10 fragment would remain soluble and spontaneously bind the ccGFP S11. The new system behaves similarly to split GFP, but develops fluorescence three-fold faster during complementation. The ccGFP is largely orthogonal to our existing split CFP and YFP variants^22^ which have recently been further characterized by Pinaud et al.^23^, and helps develop a palette of robust folding-optimized orthogonal split fluorescent proteins for multiplexing experiments.

## Results

### Engineering a monomeric and stable *Corynactis californica* GFP protein scaffold

#### Choosing a fluorescent protein

*Corynactis californica* a bright red colonial anthozoan similar to sea anemones and scleractinia stony corals, expresses several fluorescent proteins in its morphs. Schnitzler et al. identified two red fluorescent proteins^24^. One displays an as-yet-uncharacterized timer phenotype (slow conversion of chromophore from green to red) that varies according to expression conditions. The second red fluorescent protein has very poor fluorescence quantum yield. There are also a yellow and an orange fluorescent protein. Morphs of *C. californica* express at least two green fluorescent proteins. One is partially folded when expressed in *E. coli*, while the other is mostly misfolded and non-fluorescent. We chose to pursue the *C. californica* ccGFPs for three reasons. First, the multimeric red proteins appeared to have disadvantages (see above) and the yellow and orange fluorescent proteins were poorly characterized. Second, previous work by Tsien^25^ and others showed that considerable engineering may be required to retain red fluorescent phenotypes while re-engineering monomeric mutants. We have already published a superfolder monomeric RFP (sfCherry)^20^ derived from mCherry which has been used as a split protein^19^ and which we and others^21,26^ are engineering to make more efficient. Third, starting from our split GFP, we have engineered a split YFP (T203Y)^22^, as well as an efficient split CFP (Y66W) that contains several additional obligate folding mutations^22^. We chose to pursue the insoluble ccGFP variant in particular as a stringent test of our approach for engineering efficient split fluorescent proteins^1,3,22^ as well as to develop an orthogonal split fluorescent protein system for multiplex labeling in living cells.

#### Making a monomeric, cysteine-free scaffold

**Figure 1:**
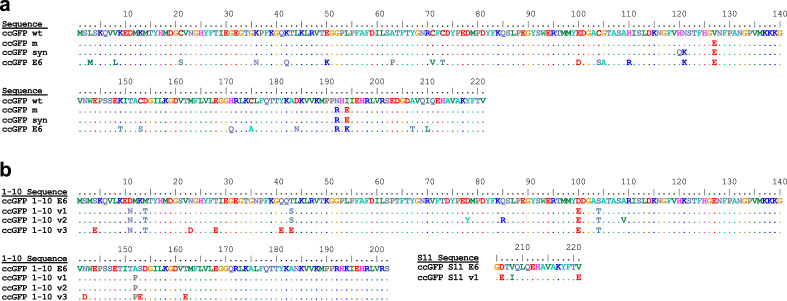
Sequence alignment of *Corynactis californica* GFPs. (**a**) Sequences leading up to the well-folded ccGFP E6. Legend: ccGFP wt, starting sequence accession number AAZ14788.1; ccGFP m, monomerizing mutations; ccGFP syn, synthetic sequence containing the monomerizing mutations and additional unexpected mutations H120Q, N121K; ccGFP E6, optimal mutant after six rounds of gene shuffling to improve folding, and three additional rounds of gene shuffling *after* replacing cysteine residues with alanine or serine (see “Methods”). (**b**) Split protein fragments for strands 1–10 and S11 used in this study, showing the starting versions derived from ccGFP E6 (see **a**) and the indicated mutants.

We posited that in order to be useful as a protein tagging and detection system, the split protein should be monomeric and have no cysteines to enable an accurate estimation of target proteins based on fluorescence signals. Predicted monomerizing mutations (V127E, N192R, I194E) were introduced to ccGFP following published protocols and based on structural homology with monomeric Azami Green^27^ (see “Methods”). The protein still failed to fold and was non-fluorescent when expressed in *E. coli*. Bright fluorescent colonies were obtained after six rounds of directed evolution using DNA shuffling (see “Methods”), converging on a small number of sequences. The brightest engineered protein retained all six native cysteine residues. Mutating the cysteines (C20S, C71A, C73S, C104S, C153S, C175A) to eliminate unwanted disulfide bond formation in the unfolded protein (or subsequent split protein fragments, *below*), resulted in misfolding and loss of fluorescence likely due to unforeseen effects on folding intermediates. After three additional rounds of directed evolution and gene shuffling, bright colonies were again obtained. The optimal final version (ccGFP E6) contains 24 mutations compared to the wild type protein (Fig. 1a): L3M, V8L, C21S, K36N, K42Q, E50K, A63P, C71V, C73T, E100D, C104S, G105A, H110R, N121K, V127E, K149T, C153S, H171Q, C105A, D184N, N192R, I194K, A207T, and I210L. Interestingly, an N121K mutation present in the template as the result of a gene synthesis error was retained. The other gene synthesis error H120Q reverted to wildtype H120. None of the amino acids replacing the six cysteines reverted to cysteine, but two had further mutated, A71V and S73T. Gel filtration chromatography confirmed the protein migrated as a monomer at ~ 15 mg/ml (Supplementary Fig. S1). ccGFP E6 was also crystallized as a monomer and its structure was successfully determined by X-ray crystallography (*manuscript in preparation*). Absorption and emission spectrum showed a strong peak at 501 nm and 520 nm, respectively (Supplementary Fig. S2).

### Engineering an efficient split system from the engineered *C. californica* scaffold

#### Improving ccGFP 1–10 and eliminating autofluorescence

**Figure 2:**
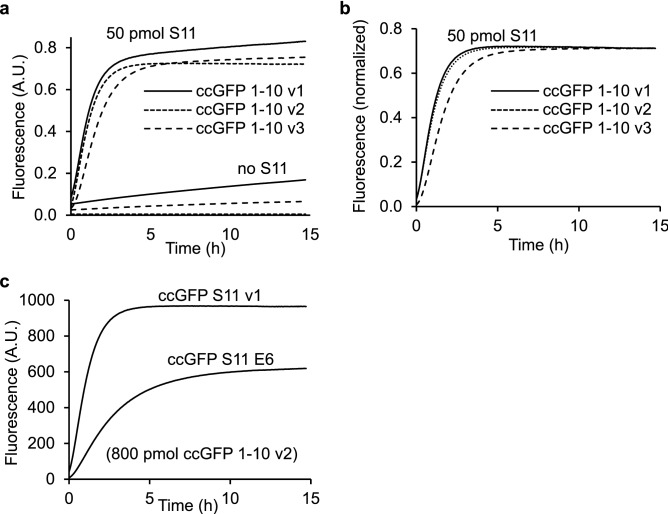
Complementation and autofluorescence of purified ccGFP 1–10 fragments. (**a**) Progress curves for in vitro complementation after mixing indicated ccGFP 1–10 variant (800 pmol) with SR-ccGFP S11 v1 (50 pmol) in 200 µl reaction wells (upper traces); development of autofluorescence of each ccGFP 1–10 without added S11 (‘no S11’, lower traces). Due to lack of chromophore residues, S11 fragments are not autofluorescent as expected (not shown). Maximum arbitrary scale signal (~ 0.8) corresponds to 45,000 fluorescence units on BioTek instrument (99,999 full scale). Progress curves were normalized by dividing measured fluorescence by the fluorescence of sfGFP control to compensate for instrument drift and jitter noise (Supplementary Fig. S8). (**b**) Normalized progress curves for indicated ccGFP 1–10 variant from (**a**) after subtraction of progress curve of corresponding ccGFP 1–10 fragments alone (‘no S11’). (**c**) In vitro complementation of equal molar amounts of ccGFP S11 variants (50 pmol) with ccGFP 1–10 v2 (800 pmol) in 200 µl reaction wells.

We followed the same strategy we used to engineer split GFP^1^. Using homology alignment with the structure (PDB 3ADF)^28^ of monomeric Azami GFP, the engineered ccGFP E6 protein scaffold was split into two pieces, the large ccGFP 1–10 E6 (amino acids 1–202, MSMSKQVLK•••RHKIEHRLVRS) and the small ccGFP S11 E6 (amino acids 205–221, GDTVQLQEHAVAKYFTV) (see also Fig. 1). Strand ccGFP S11 E6 was solubly expressed as a C-terminal tag on the carrier protein sulfite reductase^1^. The ccGFP 1–10 E6 protein aggregated when expressed alone in *E. coli* at either 37 °C or 20 °C from a pET vector, and soluble lysates did not complement with SR-ccGFP S11 E6. Directed evolution of ccGFP 1–10 (see “Methods”) dramatically improved the complementation rate and solubility. Unexpectedly, this version, termed ccGFP 1–10 v1 (Fig. 1), slowly gained fluorescence *without* the S11 fragment, (at about 1% the rate seen with excess ccGFP S11, Fig. 2a). To reduce the unwanted autofluorescence, after replating the ccGFP 1–10 library from the final round of directed evolution, we aligned images of plates after ccGFP 1–10 expression (to observe ccGFP 1–10 autofluorescence), and after SR-ccGFP S11 expression (to observe full complementation fluorescence). We identified several desirable colonies (8 out of 20,000) with ccGFP 1–10 clones that were faint or non-fluorescent alone, but that became highly fluorescent after SR-ccGFP S11 E6 expression. The best of these was isolated and termed ccGFP 1–10 v2 (Fig. 1). Relative to ccGFP 1–10 v1, ccGFP 1–10 v2 has the additional mutations D78Y, Q85R, and A109V. This variant exhibits no detectable autofluorescence (Fig. 2a).

#### Improving ccGFP S11

In our original work engineering a two-part split GFP, we found that the C-terminal GFP S11 wildtype dramatically reduced the solubility of hexulose phosphate synthase^1^ (HPS) from *P. aerophilum*, suggesting that the solubility and folding of this protein was sensitive to C-terminal split protein tags. Thus, we used HPS as ‘bait’ in a directed evolution schema in *E. coli* to discover improved mutants of ccGFP S11 for which the HPS-ccGFP S11 fusion solubility matched that of HPS alone. Libraries of ccGFP S11 variants as C-terminal fusions with HPS and ccGFP 1–10 v2 were expressed in succession in the same cells from independently inducible compatible plasmids (see “Methods”) to avoid false positives caused by cotranslational rescue of the folding of insoluble variants of HPS-ccGFP S11 that might occur with co-expressed ccGFP 1–10 v2 as previously noted for GFP^1^. The brightest clones all contained the mutations D206E, V208I, and V221E, were brighter and matured faster compared to the ccGFP S11 E6, and balanced a lack of perturbation of fusion protein solubility with good complementation (Fig. 2c). We termed this variant ccGFP S11 v1.

#### Supercharging the ccGFP 1–10 optima

ccGFP 1–10 v1 and v2 were each about 50% soluble expressed at 37 °C from pET T7 plasmids. In an attempt to increase the solubility, as had been done by others for fluorescent proteins^29,30^, we mutated some neutral or hydrophobic surface residues of ccGFP 1–10 v1 to charged residues such as Glu and Arg. The new version, ccGFP 1–10 v3, carried 8 additional negatively charged residues relative to ccGFP 1–10 v1: S4E, N23D, T28E, Q41E, S43E, N142E, S153E, T162E.

### Characterization of split ccGFP fragments by renaturation, autofluorescence, and complementation

#### Renaturation yield after unfolding

GdnHCl-denatured inclusion bodies of ccGFP 1–10 variants were renatured in 100 mM Tris, 150 mM NaCl, 10% v/v glycerol (TNG) buffer as described in “Methods”. For the same amount of inclusion bodies (~ 75 mg/tube), after dilution of the denatured inclusion bodies in 20 ml TNG, ccGFP 1–10 v1 yielded ~ 0.46 mg/ml, while ccGFP 1–10 v2 yielded ~ 0.85 mg/ml. The − 8 charged version ccGFP 1–10 v3 yielded ~ 2.5 mg/ml, a 67% yield. To facilitate comparison of specific activities of complementation with S11, for subsequent experiments, all refolded ccGFP 1–10 samples were concentrated or diluted to ~ 0.75 mg/ml.

#### Autofluorescence of ccGFP 1–10 variants

We monitored the development of autofluorescence of the ccGFP 1–10 variants alone over time. Referring to Fig. 2a, autofluorescence was significant for ccGFP 1–10 v1 and v3 but not ccGFP 1–10 v2. To test the relative in vitro complementation efficiency of the different ccGFP 1–10 variants, the same amount of SR-ccGFP S11 v1 (50 pmol) was added to a large molar excess of ccGFP 1–10 (800 pmol) (see “Methods”) (Fig. 2a). After subtraction of the blank autofluorescence progress curves as appropriate, both ccGFP 1–10 v1 and ccGFP 1–10 v2 have similar complementation kinetics, while the − 8 charged ccGFP 1–10 v3 is slower (Fig. 2b). Supplementary Fig. S4 shows the appearance of raw fluorescence progress curves for different concentrations of SR-ccGFP S11 v1 complemented with ccGFP 1–10 v3. The background autofluorescence progress curve for ccGFP 1–10 v3 could be easily subtracted. The same amount of either SR-ccGFP S11 E6 or SR-ccGFP S11 v1 (50 pmol) was added to the plate and a large molar excess of ccGFP 1–10 v2 was added (800 pmol) (see “Methods”). SR-ccGFP S11 v1 complemented significantly faster than SR-ccGFP S11 E6 (Fig. 2c).

#### Use of the split ccGFP system for in vitro protein quantification

**Figure 3:**
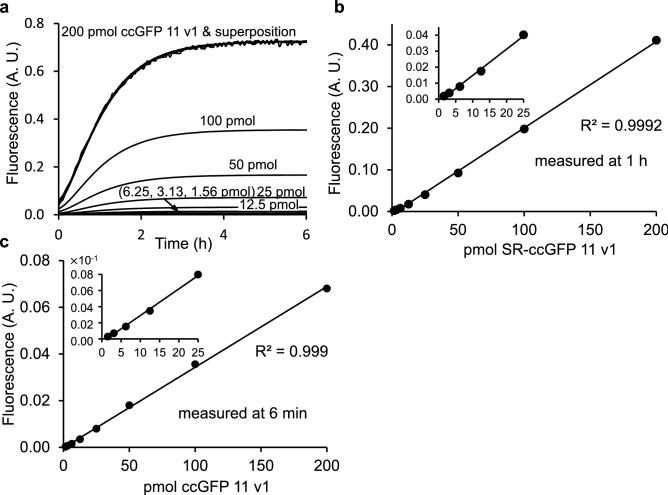
In vitro characterization of split ccGFP complementation. (**a**) Superimposition of scaled progress curves for complementation of 200, 100, 50, 25, 12.5, 6.25, 3.13 and 1.56 pmol SR-ccGFP S11 v1 in 20 µl aliquots, mixed with 180 µl aliquots containing 800 pmol of ccGFP 1–10 v2. Maximum signal (~ 0.8) corresponds to 45,000 fluorescence units on BioTek instrument scale (99,999 full scale). Progress curves were normalized by dividing measured fluorescence by the fluorescence of sfGFP control to compensate for instrument baseline drift (Supplementary Fig. S8). The curves can be superimposed by linear scaling indicating that the shape of the progress curve does not depend on the concentration of the tagged protein or depletion of the pool of unbound ccGFP 1–10 fragment. Note, in the superposition (top), noisy traces naturally result from the required scaling of the lowest concentration progress curves. (**b**) In vitro sensitivity of SR-ccGFP S11 v1 complementation with ccGFP 1–10 v2. Values of progress curves at 1 h from (**a**) are plotted vs. concentration of SR-ccGFP S11 v1. (**c**) Same as Fig. 3b, but data from (**a**) taken at 6 min.

We measured fluorescence progress curves for complementation of purified SR–ccGFP S11 v1 and ccGFP 1–10 v2 in 200 µl reactions in a microtiter plate (Fig. 3). We avoided potential higher-order kinetic effects by initiating the complementation using a high concentration and large molar-excess of ccGFP 1–10 (800 pmol). Progress curves over a wide concentration range could be superimposed by linear scaling (Fig. 3a). Over the range of S11 analyte tested (1.56–200 pmol) it was not necessary to wait until the reactions approached their asymptotic limit (~ 6 h) to generate calibration curves. For example, linear calibration curves were easily generated at 1 h (Fig. 3b), or even as soon as 6 min (Fig. 3c) after the start of complementation. Progress curves were also measured for SR-ccGFP S11 v1 vs. either ccGFP 1–10 v1 (Supplementary Fig. S3a) or ccGFP 1–10 v3 (Supplementary Fig. S4a). After subtraction of the blank progress curves due to formation of intrinsic fluorescence (no SR-ccGFP S11) (Supplementary Figures S3b, S4b), calibration curves could be generated (Supplementary Figures S3c,d, S4c,d). The efficiency of complementation was measured as a function of pH (see Supplementary Fig. S5). The complementation rate was highest above pH 7.0, decreasing linearly with decreasing pH. Below pH 5.0 complementation was inefficient. Abosrption and emission spectrum of the SR-ccGFP S11 v1 complemented with ccGFP 1–10 v3 showed a strong peak at 501 nm and 515 nm, respectively (Supplementary Fig. S6) indicating that the split ccGFP system has similar fluorescent properties compared to the full length ccGFP E6.

### Expression and solubility screens of 18 control proteins from *P. aerophilum*

**Figure 4:**
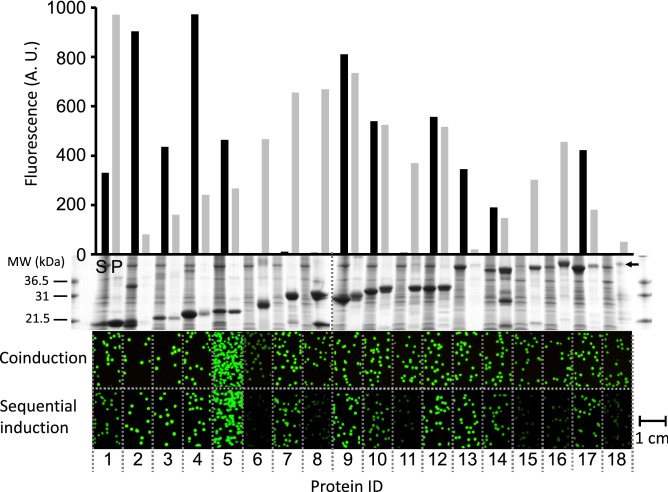
In vitro protein quantification and in vivo protein expression and solubility screens in *E. coli*. Protein quantification of eighteen *P. aerophilum* test proteins (see Supplementary Table S1) expressed as N-terminal fusions with ccGFP S11 v1 from the strong T7 promoter (bar graph, top). The ccGFP fragment complementation assay fluorescence of soluble (black bars) and unfolded pellet fractions (gray bars) using ccGFP 1–10 v2 (top). Arbitrary fluorescence units (A. U.). SDS-PAGE of the corresponding soluble (S), and pellet fractions (P) (middle). Note that protein #8, tartrate dehydratase $\beta$-subunit, shows a second lower band at ~ 13 kDa. #14, nirD protein, shows secondary bands at ~ 27 kDa and ~ 13 kDa. Original pictures of the 2 SDS-PAGE gels showing the soluble and pellet fractions of 18 test proteins are included as Supplementary Figs. S9, S10. In vivo solubility and expression screen using split ccGFP (lower). The same *P. aerophilum* test proteins cloned with a C-terminal ccGFP S11 v1 tag on tet promoter plasmid, in *E. coli* BL21 (DE3) strain carrying a pET plasmid for expression of ccGFP 1–10 v2. Fluorescence images of colonies on plates after total expression screen by coinduction of the tagged constructs and ccGFP 1–10 v2 (upper row of colonies); or after soluble expression screen by transient expression of the tagged constructs followed by expression of the ccGFP 1–10 v2 (lower row of colonies). Fluorescence images of colonies were cropped from the same pictures. Note 1 cm scale bar (lower right) illustrating the size of the colonies.

To test the utility of the split ccGFP screen for quantifying protein expression in vitro, 18 control proteins (see Supplementary Table S1) with different expression and solubility levels from *P. aerophilum*, carrying the C-terminal ccGFP S11 v1 tag, were expressed in *E. coli* at 37 °C from pET vectors using the strong T7 promoter, and split into soluble and pellet fractions. The same proteins had previously been used to test split GFP^1^, facilitating comparison with the performance of the new ccGFP (*this work*). Aliquots of the soluble fractions and solubilized denatured inclusion bodies, processed to allow direct comparison (see “Methods”) were complemented with ccGFP 1–10 v2 and the final fluorescence values were measured (Fig. 4, top). The final fluorescence was reflective of the amount of the corresponding protein in the soluble and inclusion body fractions as revealed by SDS-PAGE (Fig. 4, middle). Since several of the urea-solubilized inclusion bodies visibly aggregated soon after dilution in the assay buffer, the successful complementation implies that the ccGFP 1–10 fragment rapidly binds the S11 tag during the dilution step before the formation of insoluble aggregates, committing the chromophore to form regardless of the subsequent solubility of the complex. We previous observed rapid binding of complementary split GFP fragments^1^.

#### Estimating total protein expression in vivo in living bacterial cells

To estimate total protein expression in living *E. coli*, the C-terminally ccGFP S11 v1-tagged protein (expressed from the moderately strong AnTET regulated promoter) and the ccGFP 1–10 v2 detector protein (expressed from the very strong IPTG inducible T7 promoter) were co-expressed (see “Methods”). Referring to Fig. 4, upper row of fluorescent colonies, the fluorescence can be easily detected regardless of the solubility of the protein (as estimated from the SDS-PAGE of the soluble and pellet fractions of the same protein expressed alone from the strong T7 promoter (Fig. 4, SDS gel (middle)). As previously noted for split GFP^1^, this is consistent with a model where the 1–10 fragment can rapidly bind the S11 tag as soon as it appears in the cell, committing the complex to folding and chromophore formation regardless of the subsequent fate (soluble or aggregated) of the S11-tagged protein of interest. As expected, colonies expressing protein #6 (polysulfide reductase subunit) are fainter than colonies expressing the other 17 control proteins, because its expression leads to the accumulation of large amounts of a red product, absorbing the blue 488 nm excitation light.

#### Estimating soluble protein expression in vivo in living bacterial cells

To estimate soluble expression in an *E. coli* colony assay, the S11-tagged proteins were expressed first from the moderate AnTET regulated promoter, then the expression was shut off (see “Methods”). After resting for 1 h to allow the proteins to remain soluble or become aggregated according to their intrinsic properties, and for any remaining AnTET inducer to diffuse out, the ccGFP 1–10 v2 detector protein was then expressed from the strong T7 promoter to help insure a molar excess of the 1–10 protein. Under these conditions, the 1–10 fragment should only bind soluble and accessible S11 tagged protein molecules. Referring to Fig. 4, (*lower* row of fluorescent colonies), with the exception of protein #7, nucleoside diphosphate kinase (*see below*), the fluorescence is reflective of soluble expression as estimated from the SDS-PAGE of the soluble and pellet fractions of the same protein expressed alone from the stronger T7 promoter (Fig. 4, SDS gel (*middle*), and Supplementary Table 1). The in vivo solubility of the protein #7 is higher from the moderate AnTET regulated promoter (based on colony fluorescence, Fig. 4*bottom row of colonies*) compared to its expression from the strong T7 promoter (SDS gel lanes, Fig. 4, *middle*, and Supplementary Table 1). This is consistent with our earlier observation^1^ using SDS-PAGE that showed protein #7 tagged with GFP S11 M3 is partially soluble as expressed from the moderate pTET AnTET promoter, and insoluble expressed from pET T7 promoter^1^. Taken together, the behavior of the 18 control proteins tagged with the ccGFP S11 v1 is consistent with earlier findings with the optimal GFP S11 M3 fragment^1^, suggesting that the optimized ccGFP S11 v1 tag does not strongly perturb the solubility behavior of the fusion proteins.

### Testing cross-complementation between GFP and ccGFP split protein fragments

**Figure 5:**
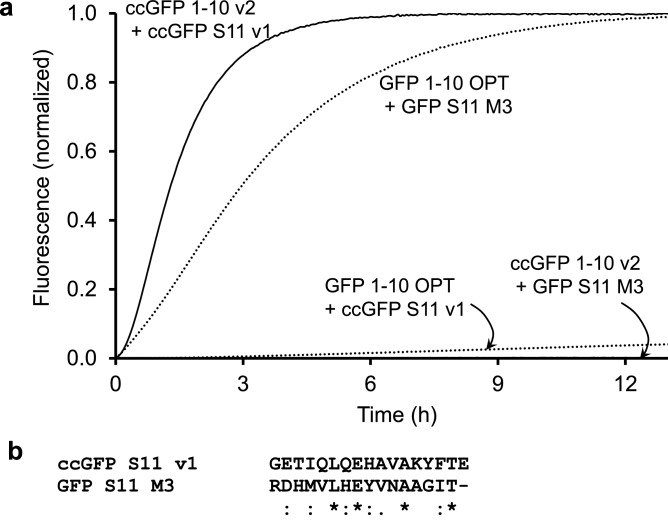
(**a**) Normalized progress curves for complementation of cognate and non-cognate ccGFP and GFP fragments. Rapid complementation of cognate fragments (upper curves). ccGFP 1–10 v2 and SR-ccGFP S11 v1 (upper solid line); GFP 1–10 OPT and SR-GFP S11 M3 (upper dotted line). GFP fragments are from the original split GFP^1^ derived from *A. victoria*. Weak complementation between non-cognate fragments (bottom curves). Complementation between ccGFP 1–10 v2 and its non-cognate fragment SR-S11 M3 is not detectable and the trace is at the baseline (bottom solid line). Complementation of GFP 1–10 OPT with the non-cognate fragment ccGFP S11 v1 (lower dotted line) is ~ 4% of the final value for the cognate GFP S11 fragment (upper dotted line). Complementation was initiated by mixing 800 pmol of each 1–10 fragment with 50 pmol of S11 fragment in 200 µl reaction wells. (**b**) Amino acid sequence alignment showing sequences of ccGFP S11 v1 and GFP S11 M3.

To test the ability of ccGFP fragments to recognize GFP fragments and vs. versa, complementation reactions were set up between the non-cognate pairs, i.e. ccGFP 1–10 v2 with GFP S11 M3^1^, and GFP 1–10 OPT^1^ with ccGFP S11 v1 (Fig. 5a). Complementation reactions were also set up between cognate pairs of fragments i.e. ccGFP 1–10 v2 with ccGFP S11 v1, and GFP 1–10 OPT with GFP S11 M3. Referring to Fig. 5a, under the conditions of the assay, the GFP 1–10 OPT fragment weakly complements with ccGFP S11 v1, yielding a fluorescence value after ~ 12 h and ~ 4% that of its cognate interaction with GFP S11 M3. Notably this reaction had not reached completion at 12 h. CFP 1–10 OPT^22^ also weakly complements with ccGFP S11 v1 but with lower efficiency compared to GFP 1–10 OPT (Supplementary Fig. S7). In contrast, under the same conditions, ccGFP 1–10 v2 does not detectably complement with GFP S11 M3 (Fig. 5a). Amino acid sequences of ccGFP S11 v1 and GFP S11 M3 were aligned with each other in Fig. 5b.

### Double-labeling experiments

**Figure 6:**
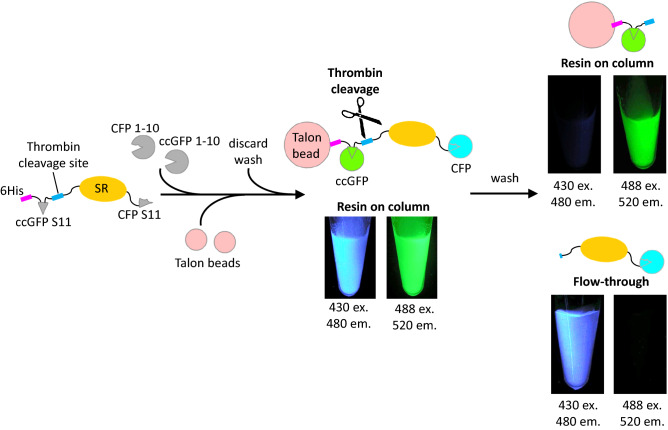
Double-labeling experiment with split CFP^22^ and ccGFP (this work). Sulfite reductase with an N-terminal 6HIS and ccGFP S11 v1 tag, and a C-terminal CFP S11 M3 tag was complemented with an excess of CFP 1–10 OPT and ccGFP 1–10 v2 (left). Talon metal affinity beads were added to capture the complex, washed and the beads imaged to show CFP and GFP bound (middle). After cleavage of the protein on the beads by added thrombin, the beads were washed and the resin and flow through imaged to reveal where the CFP and GFP localized (right). As expected, the ccGFP was retained on the beads (upper right), while the CFP was found in the flow-through (lower right).

To ascertain the utility of the new split ccGFP in cyan and green fluorescent protein double-labeling experiments, we tagged the soluble protein sulfite reductase from *P. aerophilum* with an N-terminal ccGFP S11 v1, and a C-terminal GFP S11 M3 fragment^22^. The construct had an N-terminal 6His tag for capture by metal affinity resin, followed by a thrombin tag for selective cleavage and release from the bead. Referring to Fig. 6, after mixing the tagged complex with CFP 1–10 OPT^22^ and ccGFP 1–10 v2, the fluorescent complex was captured on Talon resin. Imaging the washed beads with the appropriate excitation and emission filters revealed the expected cyan and green fluorescence. The complemented CFP was recovered in the wash after cleavage by thrombin, while the ccGFP was retained on the resin via the 6His tag (Fig. 6).

## Discussion

The optimized ccGFP 1–10 v2 contains 32 mutations relative to the wild type ccGFP protein, and 9 mutations compared to the folding optimized full-length ccGFP E6 (Fig. 1). Most of the mutations are likely required for the enhanced phenotypes, as unlike conventional error prone mutagenesis where mutants are not subjected to recombination, the DNA shuffling (fragmentation and homologous recombination of the 30 best performing clones per cycle of evolution) used here typically result in ‘backcrossing as you go’, i.e. removal of non-essential or neutral stochastic mutations and amplification of beneficial mutations as seen previously^31,32^. Optimized ccGFP S11 v1 contains three mutations relative to the starting ccGFP S11 E6 fragment (Fig. 1). These significantly improve complementation with the 1–10 fragment (Fig. 2c). The autofluorescence phenotype in ccGFP 1–10 v1 and v3 is intriguing. Apparently, the chromophore residues in the 1–10 fragment are capable of forming a population of fluorescent states even without the S11 strand. Here, we find autofluorescence of the ccGFP 1–10 v1 can be eliminated by three mutations (D78Y, Q85R, and A109V yielding ccGFP 1–10 v2) without strongly effecting complementation. Since backcrossing was not done after selecting this single triple mutant of ccGFP 1–10 v1, further work will be needed to ascertain whether all of the three mutations are required.

Various strategies have been used to apply selective pressure during the evolution of split fluorescent proteins, based on the intended application. For example, starting from a superfolder red fluorescent protein ‘sfCherry’^20^, Bo Huang et al. evolved an improved split sfCherry for CRISPR-based protein labeling^21^. Initially the authors applied selective pressure by inserting a long disrupting peptide loop between strands 10 and 11 in the full-length sfCherry prior to mutation and selection of brighter clones. When co-expressed from different constructs, the complementation of the resultant mutant sfCherry 1–10 and S11 modestly improved, likely because the peptide loop insertion did not apply sufficient selective pressure^21^. In a subsequent paper the authors co-expressed the sfCherry fragments as separate polypeptides during the mutation and selection^26^. Not surprisingly, these fragments resulted in much brighter complementation relative to the starting sfCherry fragments and appeared well-suited for co-expression in vivo, such as after CRISPR labeling. The authors also used the new split cherry in one experiment to label synapse partners^26^. Others have previously noted that versions of GFP 1–10 that are prone to aggregation when expressed alone, can be rescued when co-expressed with GFP S11^1^. Thus, split proteins evolved for co-expression may perform adequately when used for routine protein labeling in cells constitutively expressing the fragments, but may fail in the panoply of more stringent applications described in the introduction (above) where expression of the S11-tagged protein and 1–10 are separated in time and space. It remains to be seen if this is a limitation of the split sfCherry fragments of the Huang lab. Thus in the present work, and in our earlier work with split GFP^1^, we maximized selective pressure during evolution, transiently expressing the 1–10 fragment alone, and then shutting off expression to allow ample time for it to aggregate prior to subsequent expression of the S11 fragment.

We had considered starting with soluble monomeric Azami green, rather than the insoluble ccGFP used here, but decided against this as having a cysteine-free split protein system was an important goal for eliminating the possibility of disulfide formation, and Azami green contains four cysteines. We anticipated that replacing the cysteines with amino acids serine or alanine could reduce folding yield, potentially offsetting any advantages of using Azami. Indeed, after we had initially evolved a soluble ccGFP, replacing the cysteines with alanine or serine greatly reduced folding yield, and three additional rounds of mutagenic shuffling and selection were required to restore it as ccGFP E6 which is highly soluble and fluorescent expressed at 37 °C. Nonetheless, after extensive engineering of the ccGFP fragments, the resulting ccGFP 1–10 optima are ~ 50% soluble when expressed at 37 °C in *E. coli*. It is tempting to postulate that starting with a more soluble fluorescent protein, other than the initially soluble ccGFP used here, might have resulted in an even more soluble ccGFP 1–10. This is not necessarily the case. For example, in the case of sfGFP, (which is exceptionally soluble and which was specifically engineered to resist misfolding and still fluorescent even when fused to misfolding proteins), the GFP 1–10 fragment was initially mostly insoluble^1^. It required extensive evolution to make it ~ 50% soluble and capable of complementing GFP S11 *in trans*^1^. Partial solubility of ccGFP 1–10 should not preclude its use in a variety of applications. Indeed, optimized GFP 1–10 too is only ~ 50% soluble when expressed at 37°C^1^, and it is widely used by other groups in both eukaryote and prokaryote cell types (vide supra, Introduction, this manuscript). Finally, in keeping with the behavior of overexpressed proteins, both optimized GFP 1–10 and ccGFP 1–10 are more soluble expressed at lower temperatures (*data not shown*), and as illustrated in “Methods” section (vide infra, this manuscript) the inclusion bodies obtained by forcing overexpression at 37 °C can be a ready source of substantially pure 1–10 for in vitro applications after washing, unfolding in guanidine, and diluting ~ 20-fold in buffer. In the future, additional increases in solubility of the 1–10 fragments might be achieved by further increasing the stringency of selection by attaching partially soluble proteins during evolution, as was done for the S11 fragments here.

The split ccGFP 1–10 v2 and S11 v1 complement nearly threefold faster than the corresponding split GFP fragments, reaching 80% completion in 2 h rather than 6 h (Fig. 5a). When quantifying S11-tagged proteins in lysates (Fig. 4), it is not necessary to wait until the complementation reaction asymptotically approaches completion. Reproducible linear calibration curves can be generated at 1 h (Fig. 3b) or as early as 6 min (Fig. 3c). The complementation is sufficiently robust to allow quantification of solubilized inclusion bodies (Fig. 4). In living bacterial cells, transient expression of S11 tagged proteins followed by expression of the 1–10 fragment gives colony fluorescence reflective of soluble protein (Fig. 4). In contrast, co-expression of S11 tagged protein along with 1–10 produces fluorescence proportional to total expression. The results of protein solubility test for 18 control proteins from *P. aerophilum* using the split ccGFP 1–10 v2 and S11 v1are in good agreement with in vitro solubility test and with those using our previously published split GFP^1^ indicating that ccGFP 1–10 v2 is efficient for both in vivo and in vitro assays (Supplementary Table 1). The − 8 charged variant of ccGFP 1–10 v1, ccGFP 1–10 v3, yields more soluble protein compared to ccGFP 1–10 v1 during refolding (2.5 mg/ml vs. 0.85 mg/ml), and might be preferred for in vitro assays, since it is a simple matter to eliminate the interference of autofluorescence by subtracting a blank progress curve measured for the 1–10 alone (Supplementary Fig. S3). While the charged variant exhibits slightly lower autofluorescence compared to ccGFP 1–10 v1 (Fig. 2a), it complements more slowly than either ccGFP 1–10 v1 or v2 (Fig. 2). A detailed comparision of three versions of ccGFP 1–10 and their suggested uses is included in Table 1.

**Table 1:** Summary of three versions of ccGFP 1–10 and their suggested uses for in vivo and in vitro assays.

ccGFP 1-10 version	Autofluorescence (Fig. 2a)	Complementation rate (Fig. 2b)	Refolding yield (mg/ml)	In vivo assay	In vitro assay
ccGFP 1·10 v1	Yes	Fast	0.85	No	Yes
ccGFP 1-10 v2	No	Fast	0.5	Yes	Yes
ccGFP 1·10 v3	Yes	Slower	2.5	No	Yes

The new split ccGFP provides an additional detection tool to our existing split GFP, YFP, and CFP systems and has great potential for multiplexed labeling. However, while ccGFP 1–10 does not complement with GFP S11, GFP 1–10 and CFP 1–10 complement ccGFP S11 at about 4% or less the efficiency of its cognate interaction with GFP S11 (Fig. 5, Supplementary Fig. S7). Interestingly, amino acid sequence alignment of ccGFP S11 v1 and GFP S11 M3 showed ~ 25% sequence identity but over 50% sequence similarity (Fig. 5b). Despite this limitation, the strong preference for cognate interactions should facilitate a variety of double-labeling or multiplex experiments as illustrated in Fig. 6 for split CFP and ccGFP. Related work in our labs suggests it should be straightforward to mutate the interface of the ccGFP S11 so that it is no longer recognized by GFP 1–10 but still recognized by ccGFP 1–10. Future work will show whether ccGFP fragments might cross-complement with split cherry^26^.

Complementation of GFP 1–10 and S11 fragments appears to be complicated by an equilibrium between a complementation-competent monomeric 1–10 and a quiescent dimeric 1–10 that does not complement S11^23^. Early work suggested that the GFP 1–10 dimer had a lower specific activity than the monomeric species for complementation with S11^1^, but results were not conclusive since the studies involved gel-purified monomers and dimers, which likely underwent slow interconversion during subsequent experiments^1^. More recently, by detailed kinetic analyses, Pinaud and co-workers showed that only the monomeric form of GFP 1–10 appears to complement with S11^23^. Whether autofluorescence of ccGFP 1–10 v1 depends on dimerization/oligomerization of the 1–10 fragments, and whether ccGFP 1–10 exhibits the same dimer/monomer equilibrium seen for split GFP, will be the subject of future studies aimed and further understanding the ccGFP folding and assembly. ccGFP has much greater sequence homology to the tetrameric *Discosoma* sp. red fluorescent protein than to the monomeric *A. victoria* GFP^24^, which may affect its folding mechanism. Measurement of the stability of the GFP 1–10 fragments might be possible using deuterium exchange NMR spectroscopy, and stability and kinetic folding studies of the full-length ccGFPs (carrying the corresponding 1–10 v1 or v2 and S11 optima mutations) would offer insight into understanding how ccGFP v2 eliminates the autofluorescence phenotype. For example, full length ccGFP carrying the 1–10 v2 mutations might have lower thermodynamic stability compared ccGFP carrying the 1–10 v1 mutations, possibly destabilizing a folding intermediate leading to chromophore formation in the 1–10. The split ccGFP mutations might also confer exceptional stability to the full-length scaffold, as has been seen previously for engineered split fibronectin domains^33^. Experiments to measure the stability, folding kinetics, and to obtain crystal structures of the full-length variants are underway.

## Methods

### Engineering a monomeric version of ccGFP for improved solubility and folding

*Corynactis californica* GFP (ccGFP) wildtype protein was predicted to be a tetramer, predicted monomerizing mutations (V127E, N192R, I194E) were introduced following published protocols based on structural homology with monomeric Azami Green^27^. The gene ordered from Blue Heron contained two unexpected wobble mutations H120Q, N121K (CAT to CAA, and AAT to AAA, respectively). This wildtype ccGFP full length protein was evolved to improve solubility and folding by directed evolution. The DNA coding ccGFP was PCR amplified using vector flanking primers and was subjected to DNA fragmentation and shuffling using published protocols^1^. The library of DNA plasmids was transformed into *E. coli* BL21 (DE3) gold (Novagen) competent cells for protein expression. The 1.0 OD_600nm_ cell stock frozen library was diluted by two sequential 400-fold dilutions and 600 µl plated on each of 5 supported nitrocellulose membranes resting on 150 mm dia. Bauer plates containing Luria–Bertani (LB) agar supplemented with 50 µg kanamycin/ml media as previously published^1^. Cells were grown overnight at 32 °C and proteins were expressed by transferring the membrane colony side up to LB agar plates containing 50 µg kanamycin/ml media and 1 mM isopropyl-β-d-1-thiogalactopyranoside (IPTG) for 3 h at 37 °C. Clones (~ 30 to 40) displaying the brightest fluorescence (488 nm excitation/520 nm emission) were selected, grown overnight and frozen as 20% glycerol/LB freezer stocks at − 80 °C. Pooled plasmid preps of these clones served as templates for PCR for the next round of evolution. After six rounds of directed evolution, sequences of the brighter 30–40 constructs were confirmed by DNA sequencing and the brightest clone was chosen for subsequent modification. The six cysteine residues were mutated to alanine or serine by primer-directed PCR (C20S, C71A, C73S, C104S, C153S, C175A). The protein was subjected to another three rounds of directed evolution using the same protocol as indicated and the final, brightest, monomeric clone named ccGFP E6 was chosen for engineering the split version of the protein.

### Engineering an efficient split ccGFP 1–10

Using ccGFP E6 as starting scaffold, protein was first split into two fragments: ccGFP S11 E6 (amino acids 205–221, GDTVQLQEHAVAKYFTV) and ccGFP 1–10 E6 (amino acids 1–202, MSMSKQVLK•••RHKIEHRLVRS). ccGFP 1–10 E6 aggregated and was primarily found in inclusion bodies. Neither refolded inclusion bodies nor soluble cell lysates fractions could associate with ccGFP S11 E6 to form a full length, fluorescent protein. ccGFP 1–10 E6 was engineered using directed evolution as previously published and described^1^ with the following modifications. Briefly, the DNA library of shuffled ccGFP 1–10 was ligated into our in-house engineered^1^ pTET-SpecR with ColE1 origin, (which expresses the tetR regulator), where expression is regulated by addition of anhydrotetracycline (AnTET). The plasmid library was transformed into *E. coli* BL21 (DE3) gold (Novagen) competent cells containing the sulfite reductase–ccGFP S11 E6 tagged protein on a modified p15A vector with kanamycin resistance marker^1^ where the protein of interest is inducible with IPTG. The 1.0 OD_600nm_ cell stock frozen library was diluted by two sequential 400-fold dilutions and 600 µl plated on each of 5 supported nitrocellulose membranes resting on 150 mm dia. Bauer plates containing Luria–Bertani (LB) agar supplemented with 50 µg kanamycin/ml and 50 µg spectinomycin/ml as previously published^1^. Cells were grown overnight at 32 °C to keep colony sizes below 0.5 mm diameter, and proteins were expressed by transferring the membrane colony side up to an LB agar plate containing 50 µg kanamycin/ml*,* 50 µg spectinomycin/ml and 350 ng/ml AnTET for 3 h at 37 °C to express the ccGFP 1–10 library. The membrane was then transferred to an agar plate containing only kanamycin and spectinomycin for 1 h to allow the AnTET to diffuse out of the colonies to shut off expression of the ccGFP 1–10 library. This strategy allows each mutant protein to either remain soluble, or aggregate according to their inherent propensity. The membrane was then moved onto a new LB/agar plate containing the same antibiotics plus 1 mM IPTG to induce expression of the SR-ccGFP S11 E6 for complementation of any soluble (mutant) ccGFP 1–10. Clones exhibiting the most rapid development of fluorescence, indicating soluble and functional ccGFP 1–10 mutants, were selected and stored as freezer stocks at − 80 °C in 20% glycerol/LB medium. Brighter clones were grown in 1 ml LB liquid culture containing 50 µg kanamycin/ml*,* 50 µg spectinomycin/ml to 0.3 OD_600nm_ and induced with 350 ng/ml AnTET for 3 h at 37 °C to express just the ccGFP 1–10, and after sonication and centrifugation, 20 µl of the 350 µl soluble cell extract fractions were screened for better complementation efficiency in an in vitro assay with an excess amount of purified SR-ccGFP S11 E6. The best 30 candidates were pooled, plasmid prepared, then PCR-amplified using flanking primers in the vector backbone surrounding the ccGFP 1–10 insert, and subjected to three additional rounds of directed evolution. Mutations of the top 24 optima were confirmed by DNA sequencing, revealing the population had converged on a small subset of solutions. All the brightest optima showed some autofluorescence (development of fluorescence in the absence of ccGFP S11 E6). The brightest and fastest version was termed ccGFP 1–10 v1. To eliminate the autofluorescence, the top pool from the final round was replated, and imaged after ccGFP 1–10 expression but prior to ccGFP S11 expression, and imaged again after ccGFP S11 expression. After alignment of the images in NIH Image, the brightest clones *also* exhibiting the largest dynamic range (faintest prior to, and brightest after ccGFP S11 expression) were picked and sequenced. One such clone, ccGFP 1–10 v2, was essentially non-fluorescent after expression alone. Using alignment with the structural homolog monomeric Azami Green^27^, GFP 1–10 v3, with a net − 8 charge relative to ccGFP 1–10 v1, was made by primer-directed mutagenesis, mutating neutral surfaces residues predicted to be on the surface of the protein to charged residues such as glutamate and aspartate: S4E, N23D, T28E, Q41E, S43E, N142E, S153E, T162E.

### Engineering an efficient ccGFP S11

The ccGFP S11 E6 fragment was expressed as a C-terminal fusion with the ‘bait protein’ hexulose phosphate synthase (HPS) (see Supplementary Table S1), (previously shown to become less soluble with various split fluorescent protein fragments as C-terminal fusions^1^) as HPS-ccGFP S11 E6, and ligated into our in-house engineered^1^ pTET-SpecR with ColE1 origin, (which expresses the tetR regulator), where expression is regulated by addition of AnTET. HPS-ccGFP S11 fusions were amplified by PCR and shuffled using published protocols^1^. For each round of directed evolution, the library of HPS-ccGFP S11 (containing mutants of ccGFP S11) was transformed into *E. coli* BL21 (DE3) gold (Novagen) competent cells expressing the ccGFP 1–10 v2 protein on a modified p15A vector with kanamycin resistance marker^1^ where the protein of interest is inducible with IPTG. Optima were screened in vivo using a sequential induction protocol as previously published^1^. Briefly, the 1.0 OD_600nm_ cell stock frozen library was diluted by two sequential 400-fold dilutions and 600 µl plated on each of 5 supported nitrocellulose membranes resting on 150 mm dia. LB agar Bauer plates supplemented with 50 µg kanamycin/ml and 50 µg spectinomycin/ml as previously published^1^. Cells were grown overnight at 32 °C to keep colony sizes below 0.5 mm diameter, and proteins were expressed by transferring the membrane colony side up to an LB agar plate containing 50 µg kanamycin/ml*,* 50 µg spectinomycin/ml and 350 ng/ml AnTET for 3 h at 37 °C to express the HPS-ccGFP S11 library. Each membrane was then transferred colony side up to an agar plate containing 50 µg kanamycin/ml*,* 50 µg spectinomycin/ml for 1 h to allow the AnTET to diffuse out of the colonies, shutting off expression of the HPS-ccGFP S11. This sequential strategy allowed any mutants that interfered with the solubility of the HPS to become insoluble prior to expression of the ccGFP 1–10 in the next step. Next membranes were moved colony side up to an LB agar plate containing 50 µg kanamycin/ml*,* 50 µg spectinomycin/ml and 1 mM IPTG for 2 h to induce expression of the complementary ccGFP 1–10 v2 from the pET plasmid. Brighter clones were selected, grown at 37 °C to 0.3 OD_600nm_ in 1 ml LB liquid cultures containing 50 µg kanamycin/ml and 50 µg spectinomycin/ml, and induced at 37 °C for 3 h with 350 ng/ml AnTET only to express just the HPS-ccGFP S11. Note that the medium must not contain lactose, and the cultures should not be allowed to overgrow prior to AnTET induction, otherwise the LacUV/T7 expression of the unwanted ccGFP 1–10 from the second plasmid may leak. After sonication and centrifugation, 20 µl of the 350 µl soluble cell extract fractions (estimated to contain less than 30 pmol of HPS-ccGFP S11) were screened for better complementation efficiency in an in vitro plate assay using a 96-well plate fluorimeter^1^ with a molar excess of refolded ccGFP 1–10 v2 (180 µl of 0.75 mg/ml refolded ccGFP 1–10, ~ 800 pmol). Clones with the fastest complementation rates were selected, pooled, plasmid prepped, and subjected to the next round of evolution and screening. After two rounds of directed evolution ccGFP S11 v1 was selected. HPS-ccGFP S11 v1 solubility was improved relative to HPS-ccGFP S11 E6, and HPS-ccGFP S11 v1 complemented with ccGFP 1–10 v2 three-fold faster and brighter than HPS-ccGFP S11 E6 for comparable amounts of soluble fusion protein.

### Expression and refolding of ccGFP 1–10 and GFP 1–10 fragments

Different versions of ccGFP 1–10 and GFP 1–10 proteins were expressed and prepared as previously described^1^. One liter LB cultures of BL21(DE3) cells expressing ccGFP 1–10 or GFP 1–10 constructs were grown until an OD_600nm_ of 0.5–0.7 was reached, protein expression was induced with 1 mM IPTG and cells were harvested after 5 h of induction at 37 °C. The cell pellets were resuspended in TNG buffer and lysed by sonication on ice. Inclusion bodies containing were obtained by centrifugation at 20,000*g* for 30 min, washed, and aliquoted to 75 mg per 1.8 ml eppendorf tube as previously described^1^. To prepare 25 ml of ccGFP 1–10 or GFP 1–10 assay solution, one vial containing 75 mg of prepared inclusion body was unfolded with 1 ml of 9 M urea in TNG buffer, centrifuged at 15,000*g* for 10 min, and ~ 1 ml soluble fraction was rapidly diluted by adding 25 ml of TNG buffer. Refolded protein samples were centrifuged at 15,000*g* for 10 min to remove crude precipitate (minor fraction) and the soluble solutions were filtered through a 0.2 mm syringe filter and quantified using the Bio-Rad Protein Assay Reagent Kit (Bio-Rad). To insure ccGFP 1–10 proteins were at equal concentration for subsequent experiments, protein samples were concentrated using a tangential flow Amicon Ultra-15 centrifugal filter device (10 kDa cutoff; Millipore) and protein concentrations were measured and diluted to a final concentration of ~ 0.75 mg/ml. To examine pH dependence of the complementation of ccGFP 1–10 v2 with ccGFP S11 v1, refolded ccGFP 1–10 v2 was dialyzed against various buffers at different pH values. The protein samples at different pH were then collected, concentrated and diluted to a final concentration of ~ 0.75 mg/ml. In vitro complementation assays were set up as described below and final fluorescence values were recorded using a Tecan Microplate Fluorescence Reader (Tecan).

### Expression and purification of sulfite reductase-ccGFP S11 fusion protein

Sulfite reductase (SR) from *P. aerophilum* was cloned and expressed as an N-terminal fusion with ccGFP S11 E6 WT and ccGFP S11 v1 fragments in a pET vector with a N-terminal His_6_ tag. Briefly, 1 L cultures of BL21(DE3) cells expressing SR with different versions of ccGFP S11 were grown to OD_600nm_ ~ 0.5 to 0.7 and induced with 1 mM IPTG for 4 h at 37 °C. Cells were harvested and resuspended in TNG buffer and lysed by sonication on ice for 10 min at 70% duty cycle. Cell lysates were then centrifuged at 15,000*g* for 30 min at 10 °C to remove cell debris and the supernatant was incubated with pre-equilibrated Talon^®^ cobalt metal affinity resin (Clontech) at room temperature (22 °C) with gentle rocking for 1 h to allow proteins to bind to resin. The proteins bound to resin was separated from unbound protein by centrifuging 3000*g* for 5 min, and the pelleted resin was washed three times with tenfold volume excess TNG buffer before it was packed into a gravity-flow column. The column then was washed with 50 ml of TNG buffer followed by 50 ml of TNG buffer with 5 mM imidazole, and 20 ml of TNG buffer with 20 mM imidazole to remove unbound and non-specifically bound proteins, respectively. The purified proteins were completely eluted with 30 ml of 150 mM imidazole in TNG buffer and protein solutions were dialyzed against TNG buffer to eliminate imidazole. Protein was quantified using the Bio-Rad Protein Assay reagent Kit (Bio-Rad) and was concentrated to a final concentration of 5 mg/ml using a tangential flow Amicon Ultra-15 centrifugal filter device (10 kDa cutoff; Millipore).

### In vitro complementation assays

In vitro complementation assays of various combinations of the ccGFP S11 and ccGFP 1–10 were done using previously described protocols^1^. Briefly, a 96-well white microplate (Nunc-Immuno plate, Nunc) was first blocked with 0.5% bovine serum albumin (BSA) in TNG for 10 min. Purified SR-ccGFP S11 was subjected to twofold serial dilutions in the same buffer so that the dilutions spanned the range 1.56–200 pmol per 20 µl aliquot. Protein aliquots were added to 96-well plates and complementation was performed using a large excess of ccGFP 1–10 (0.75 mg/ml, 800 pmol) added in a 180 µl aliquot. Fluorescence kinetics (488 nm excitation/520 nm emission) were monitored with a Tecan Microplate Fluorescence Reader at 3 min intervals for 15 h. The background fluorescence of a blank sample (20 µl of 0.5% BSA in TNG buffer, 180 µl of 0.75 mg/ml ccGFP 1–10 in TNG buffer) was subtracted from the final fluorescence values. For assays involving the autofluorescent ccGFP 1–10 variants v1 and v3, the progress curve for the blank (ccGFP 1–10 alone, no S11) was subtracted from the progress curve of the analytical sample, for the entire time span. For determining solubility levels of 18 protein controls, 20 µl soluble supernatant, or 10 µl of denatured unfolded inclusion bodies were added to the bottom of a Nunc assay plate. 180 µl of ccGFP 1–10 was added to complete the complementation and final fluorescence values were measured after incubation at room temperature (22 °C) overnight using the Tecan Microplate Fluorescence Reader.

### In vivo complementation assays

18 protein controls from *P. aerophilum* were cloned into the N6His pTET ColE1 AnTET vector as an N-terminal fusion with ccGFP S11 v1 fragment and transformed into BL21 (DE3) competent cells containing pET ccGFP 1–10 T7 p15 plasmid for in vivo testing as previously described for split GFP^1^. Proteins expressed from the pTET vector carry an N-terminal 6His tag and C-terminal ccGFP S11 v1 tag. In vivo protein expression and solubility screens were performed as previously described^1^. 1 OD_600nm_ frozen cell stocks in 20% glycerol LB were thawed and diluted 400-fold (twice) in LB and plated onto a nitrocellulose membrane with selective LB agar containing 50 μg/ml kanamycin and 75 μg/ml spectinomycin (the selective media). After overnight growth at 32 °C, the membrane was transferred to a pre-warmed selective media plate containing 600 ng/ml AnTET, 1 mM IPTG for 4 h at 37 °C for total protein expression screening. Under these conditions, both the S11-tagged protein and the ccGFP 1–10 detector fragment are co-expressed. The ccGFP 1–10 can rapidly bind to the S11 tag, prior to insoluble target proteins aggregating, committing to chromophore formation and leading to complementation reflective of total protein. For protein solubility testing, the membrane was transferred to a pre-warmed selective media plate containing 300 ng/ml AnTET for 2 h, transferred colony side up to its original LB selective media plate for 1 h to allow the AnTET to diffuse out, and followed by induction on an LB selective plate with 1 mM IPTG at 37 °C for 1 h to induce the ccGFP 1–10 v2. Under these conditions, the S11-tagged protein is free to remain soluble or aggregate according to its innate propensity prior to the subsequent induction of the ccGFP 1–10 detector. Thus, the fluorescence reflects soluble S11-tagged protein rather than total S11-tagged protein. The induced plates were illuminated and imaged using an Illumatool Lighting System (LightTools Research) equipped with a 488 nm/520 nm excitation/emission filters.

### Double-labeling experiments

The soluble SR protein from *P. aerophilum* was cloned and expressed with an N-terminal ccGFP S11 v1 followed by a Thrombin cleavage site, and a C-terminal GFP S11 M3 fragment^22^ in a pET vector with a N-terminal His_6_ tag. The fusion protein was expressed and purified as described above for SR-ccGFP S11 protein. The purified protein was double labeled by incubating with an excess amount of CFP 1–10 OPT^22^ and ccGFP 1–10 v2 overnight at 4 °C. Protein mixtures were then centrifuged at 15,000*g* for 10 min at 10 °C to remove any debris and the supernatant was incubated with pre-equilibrated Talon^®^ cobalt metal affinity resin (Clontech) at room temperature (22 °C) with gentle rocking for 1 h to capture protein complex on the resin. The protein complex bound to resin was separated from the excess, uncomplemented ccGFP 1–10 v2 and CFP 1–10 OPT proteins by centrifuging 7000*g* for 5 min, and the pelleted resin was washed five times with threefold volume excess of TNG buffer. Thrombin cleavage reaction was set up at 37 °C with gentle rocking for 3 h and the flow thru fraction was collected. The resin was washed three times with threefold volume excess of TNG buffer and photos were taken for both the flow thru and the protein complex remained on the resin fractions using an Illumatool Lighting System equipped with a 488 nm/520 nm and 430 nm excitation/480 nm excitation/emission filters.

## Supplementary Information

Supplementary Information.
