# Supplementary material for: Engineering an efficient and bright split Corynactis californica green fluorescent protein
Source: Sci Rep. 2021 Sep 16;11:18440. doi: 10.1038/s41598-021-98149-8 (PMC8445986; doi:10.1038/s41598-021-98149-8)
Supplement: Supplementary file 1 — Supplementary Information. [file 41598_2021_98149_MOESM1_ESM.pdf]

Engineering an Efficient and Bright Split *Corynactis Californica* Green Fluorescent Protein

Supplementary Information

Hau B. Nguyen[1,*], Thomas C. Terwilliger[1,2] and Geoffrey S. Waldo[1,*]

[1]Bioscience Division, MS M888, Los Alamos National Laboratory, Los Alamos, NM 87545, USA.

[2]New Mexico Consortium, 100 Entrada Dr, Los Alamos, NM 87544, USA

*Corresponding authors: hau@lanl.gov or waldo@lanl.gov

Supplementary Figure S1. Gel filtration data for ccGFP E6

Supplementary Figure S2. Absorption and emission spectra of ccGFP E6

Supplementary Figure S3. Progress curves, background-subtracted progress curves, and fluorescence vs. concentration calibration plots for ccGFP 1-10 v1 complemented with SR-ccGFP S11 v1.

Supplementary Figure S4. Progress curves, background-subtracted progress curves, and fluorescence vs. concentration calibration plots for ccGFP 1-10 v3 complemented with SR-ccGFP S11 v1.

Supplementary Figure S5. pH dependence of complementation of ccGFP 1-10 v2 with SR-ccGFP S11 v1.

Supplementary Figure S6. Absorption and emission spectra of split ccGFP.

Supplementary Figure S7. Complementation efficiency of GFP 1-10 OPT and CFP 1-10 OPT with the non-cognate fragment ccGFP S11 v1.

Supplementary Figure S8. Fluorescence photobleaching of sfGFP used as reference in complementation kinetic experiments.

Supplementary Figure S9. Original picture of the SDS-PAGE for *P. aerophilum* test proteins #1 to #8.

Supplementary Figure S10. Original picture of the SDS-PAGE for *P. aerophilum* test proteins #9 to #18.

Supplementary Table S1. Mass, and fraction soluble as a function of tag used for the 18 control proteins used in this study.

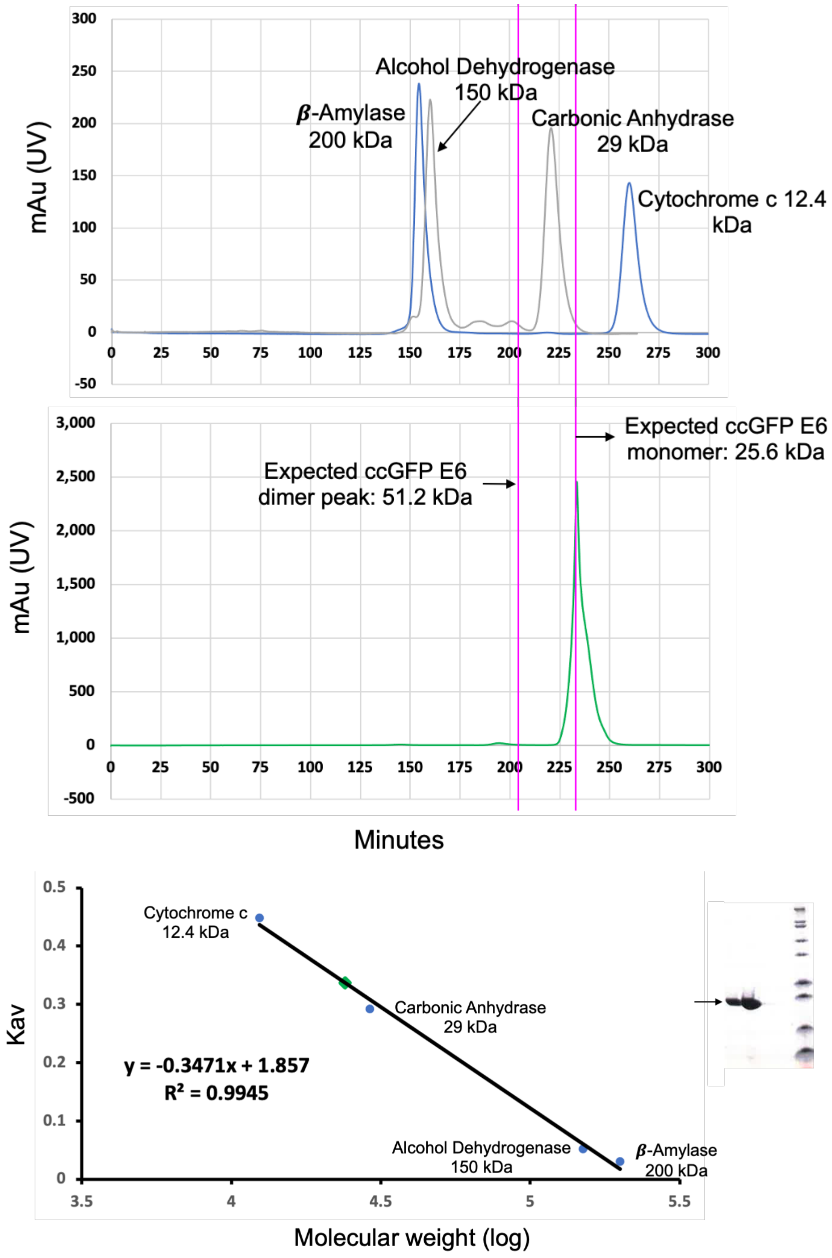

Supplementary Figure S1. Gel filtration chromatography using HiLoad Superdex 16/600 75 pg showed ccGFP E6 protein run as a monomer with calculated Mw ~24 kDa based on standard calibration curve (theoretical molecular weight is ~ 25.6 kDa including His tag). Kav = (Ve - Vo)/(Vt - Vo) where Ve = elution volume, Vo = column void volume (44.07 mL based on Blue dextran elution volume), Vc = total bed volume (120 mL). Solid line is the calibration curve calculated from the data for protein standards (R^2 = 0.9945), green square represents the main peak of ccGFP E6. Protein standards were purchased from Sigma-Aldrich (Catalog # MWGF200). ccGFP E6 has a shoulder peak with smaller Mw ~ 21 kDa that might account for some weak interaction with the resin. SDS-PAGE gel showed protein sample run as one single band.

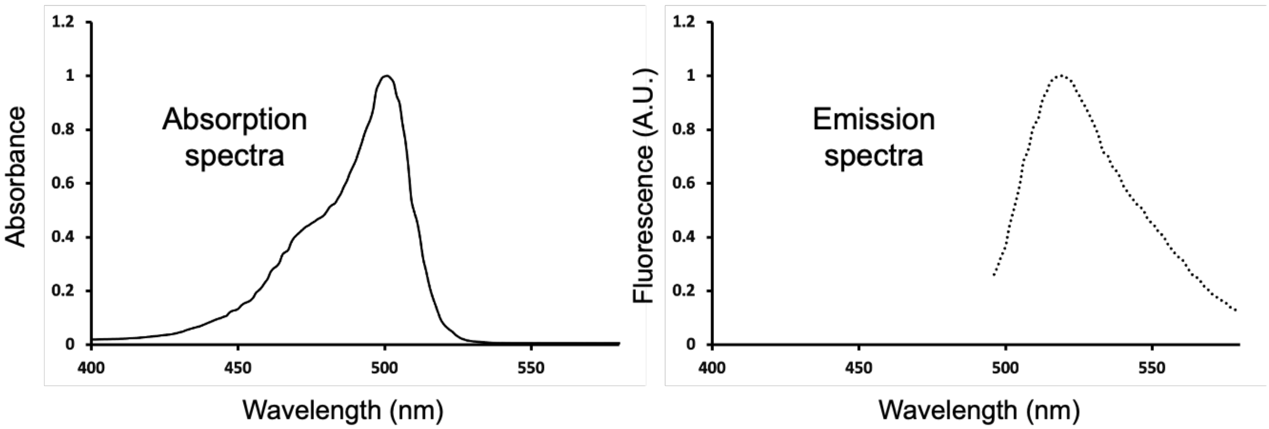

Supplementary Figure S2. Absorption and emission spectra of ccGFP E6 (excitation maximum: 501 nm; emission maximum: 520 nm)

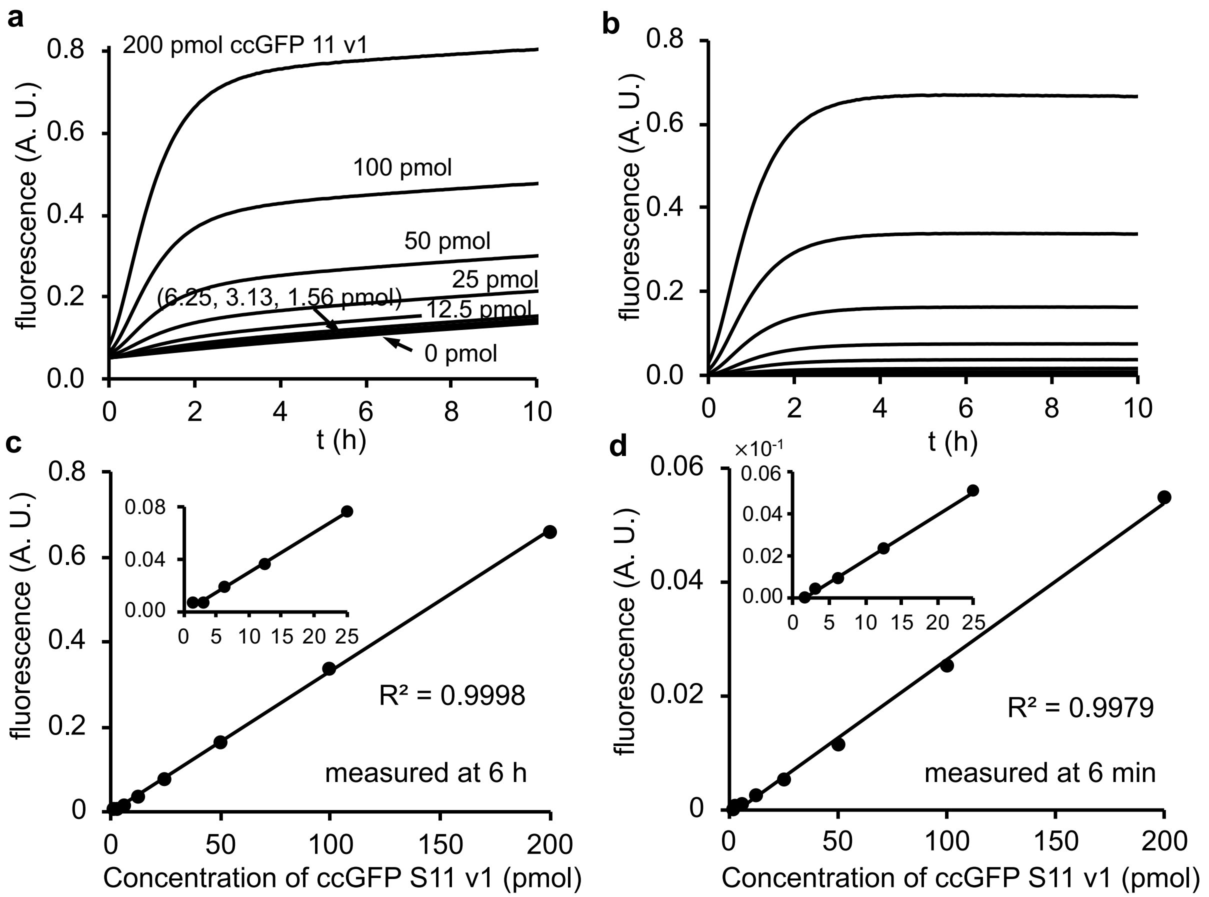

Supplementary Figure S3. (**a**) Progress curves for complementation of 200, 100, 50, 25, 12.5, 6.25, 3.13 and 1.56 pmol SR-ccGFP S11 v1 in 20 µl aliquots, mixed with 180 µl aliquots containing 800 pmol of high autofluorescence variant ccGFP 1-10 v1 in 200 µl reaction wells. (**b**) Same progress curves in (**a**) after subtraction of the blank progress curve (labeled 0 pmol) in (**a**). (**c**) *In vitro* sensitivity of SR-ccGFP S11 v1 complementation with ccGFP 1-10 v1 after background subtraction, that is the 6 h data in (**b**). (**d**) Same as (**c**), but data taken from (**b**) at 6 min.

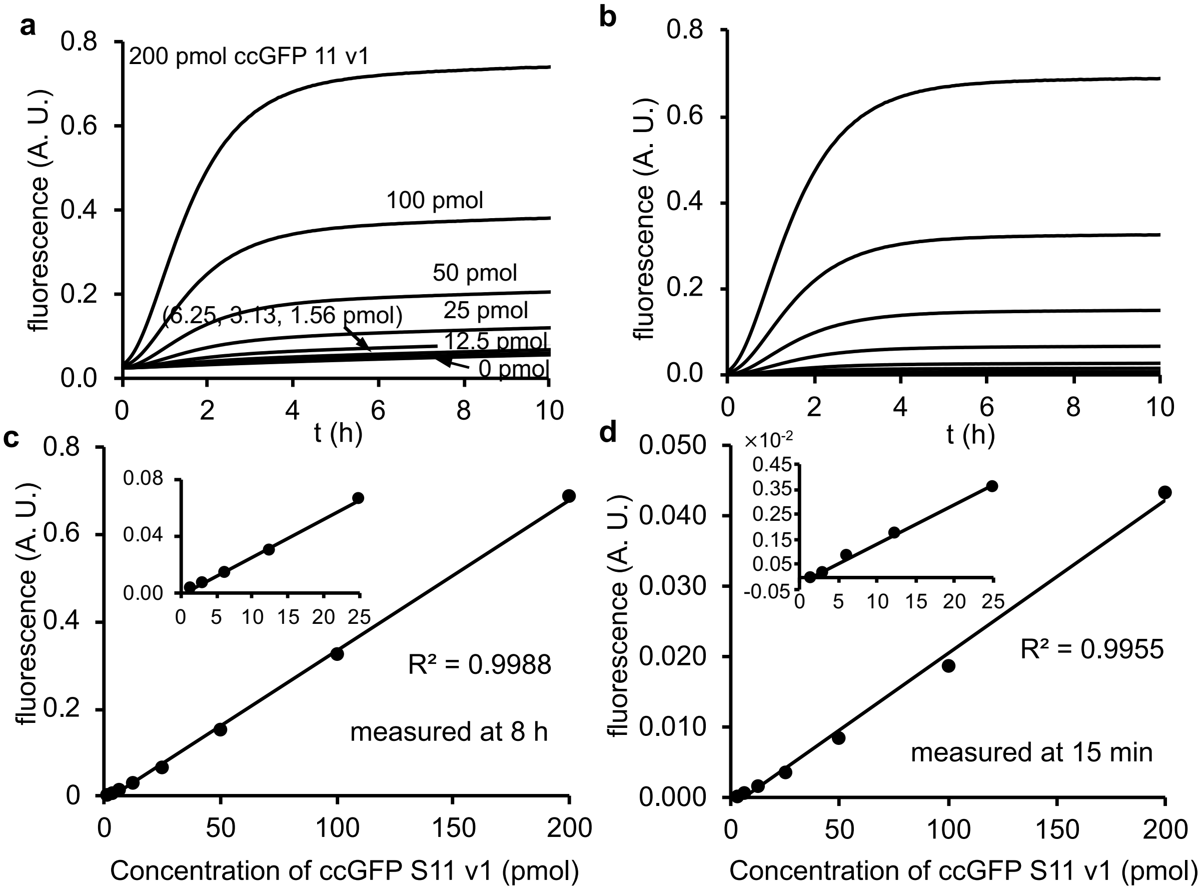

Supplementary Figure S4. (**a**) Progress curves for complementation of 200, 100, 50, 25, 12.5, 6.25, 3.13 and 1.56 pmol SR-ccGFP S11 v1 in 20 µl aliquots, mixed with 180 µl aliquots containing 800 pmol of -8 charged ccGFP 1-10 v3 in 200 µl reaction wells. (**b**) Same progress curves in (**a**) after subtraction of the blank progress curve (labeled 0 pmol) in (**a**). (**c**) *In vitro* sensitivity of SR-ccGFP S11 v1 complementation with ccGFP 1-10 v3 after background subtraction, that is the 8 h data in (**b**). (**d**) Same as (**c**), but data taken from (**b**) at 15 min.

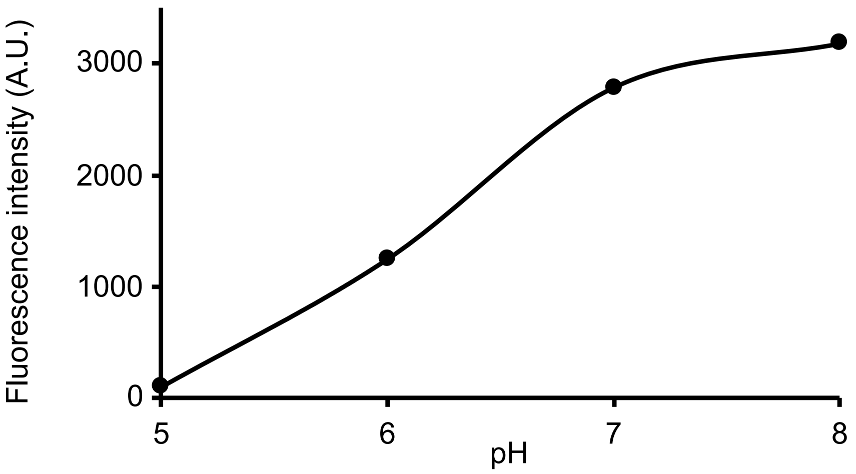

Supplementary Figure S5. pH dependence of the complementation of ccGFP 1-10 v2 with ccGFP S11 v1. Reactions initiated by mixing 25 pmol SR-ccGFP S11 v1 in 20 µl aliquot with 180 µl aliquot containing 800 pmol of ccGFP 1-10 v2, each diluted in the appropriate pH buffer (see Methods).

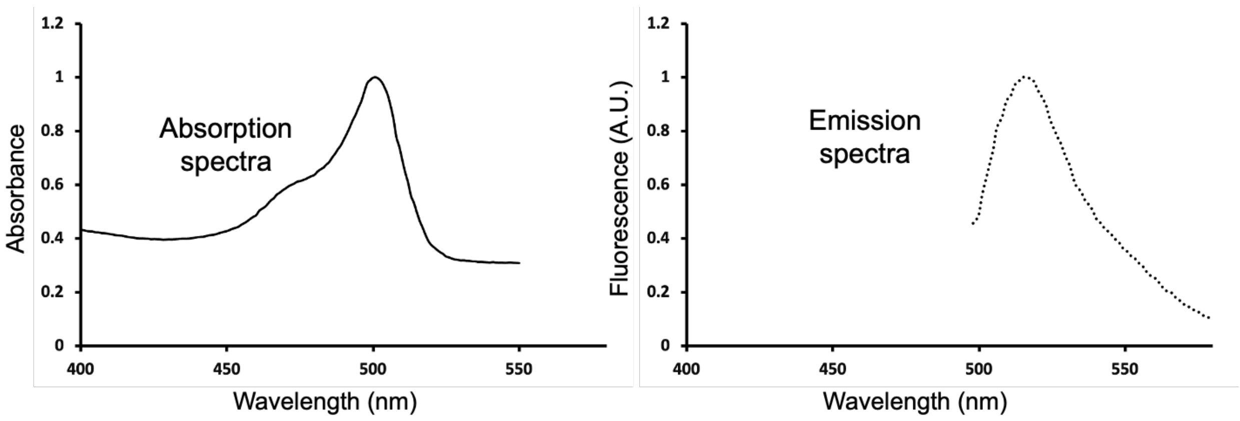

Supplementary Figure S6. Absorption and emission spectra of split ccGFP: ccGFP 1-10 v3 complemented with ccGFP S11 v1 (excitation maximum: 501 nm; emission maximum: 515 nm)

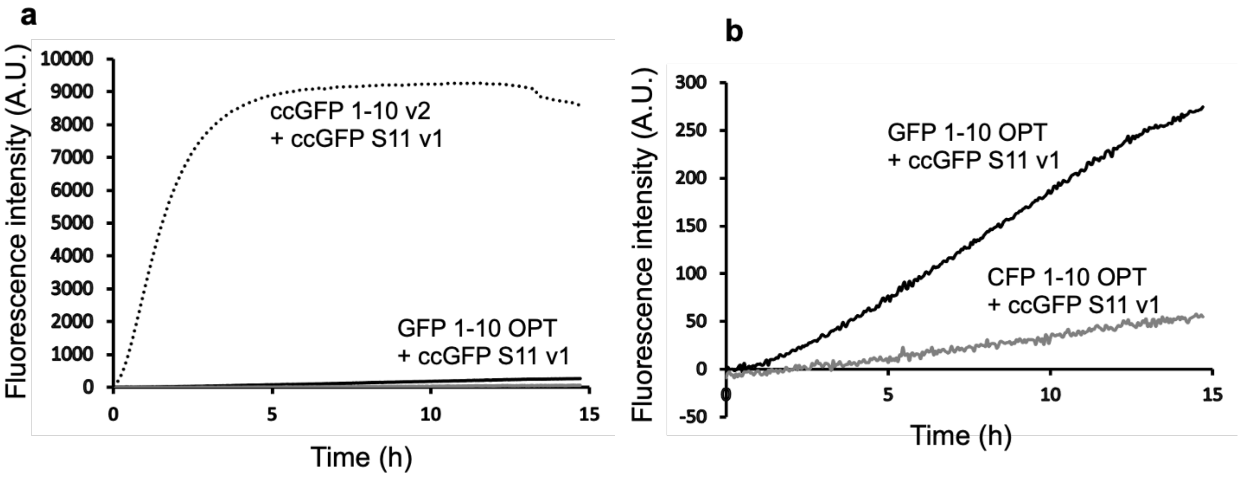

Supplementary Figure S7. (a) Progress curves (raw data) for complementation of ccGFP 1-10 v2 (dotted line), GFP 1-10 OPT (black line) and CFP 1-10 OPT with ccGFP S11 v1 (gray line). **(b)** Progress curves (raw data) for complementation of GFP 1-10 OPT (excitation: 488 nm, emission: 520 nm) and CFP 1-10 OPT (excitation: 433 nm, emission: 483 nm) with the non-cognate fragment ccGFP S11 v1 showing a tiny portion of CFP 1-10 OPT cross-react with ccGFP S11 v1. Complementation was initiated by mixing 800 pmol of each 1-10 fragment with 200 pmol of S11 fragment in 200 µl reaction wells.

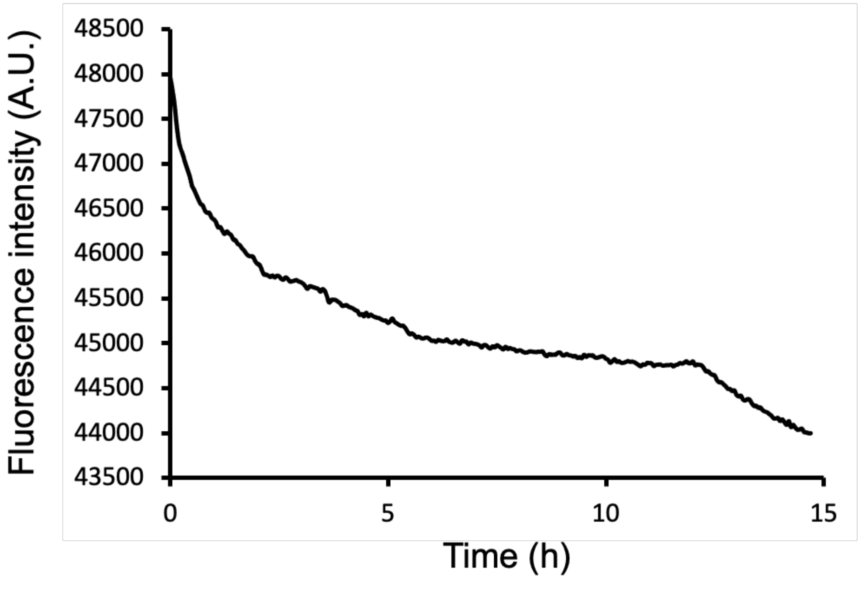

Supplementary Figure S8. Fluorescence photobleaching of sfGFP used as reference in complementation kinetic experiments (488 nm excitation, 520 nm emission)

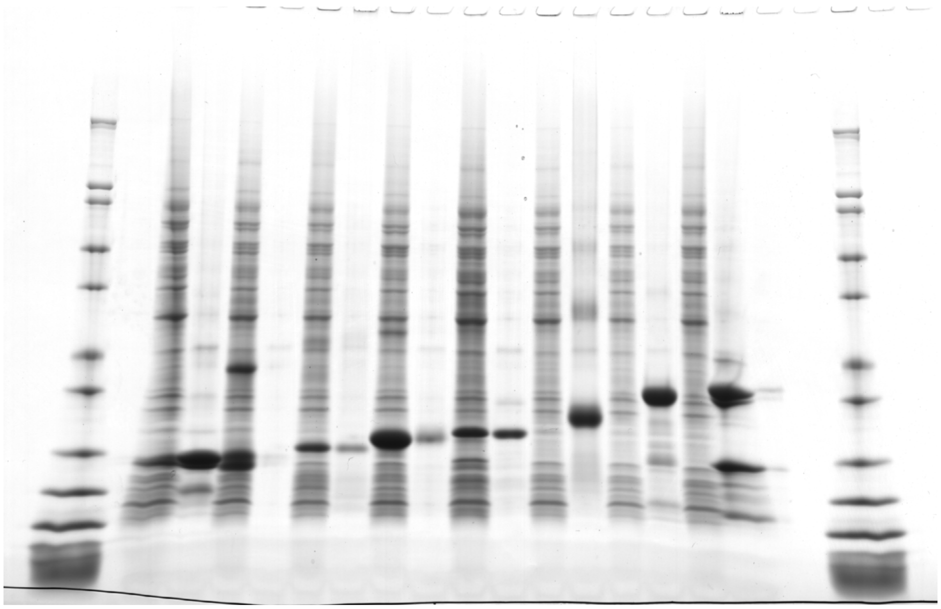

Supplementary Figure S9. Original picture of the SDS-PAGE of the corresponding soluble and pellet fractions for *P. aerophilum* test proteins #1 to #8 before being cropped as shown in Figure 4 (middle part).

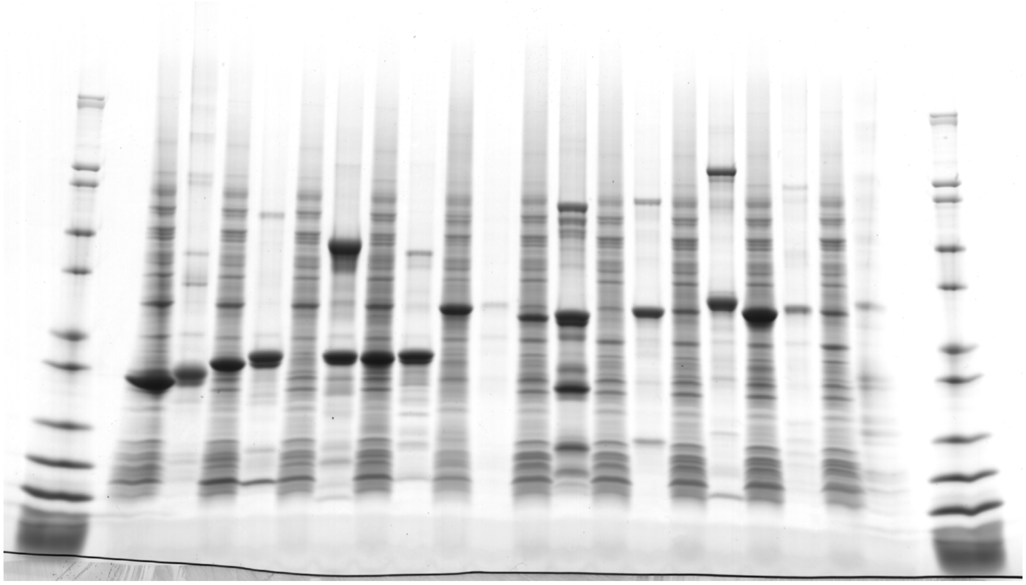

Supplementary Figure S10. Original picture of the SDS-PAGE of the corresponding soluble and pellet fractions for *P. aerophilum* test proteins #9 to #18 before being cropped as shown in Figure 4 (middle part).

Supplementary Table S1. Effect of split fluorescent protein S11 tags on the solubility of eighteen proteins from *Pyrobaculum aerophilum*.

#	[b]Protein	[c]MW	Fraction soluble		
			[d]NF	[e]GFP S11 M3	[f]ccGFP S11 v1
1	DNA-directed RNA polymerase	12.5	0.05	0.10	0.10
2	Sulfite reductase (dissimilatory subunit)	12.7	1.00	1.00	1.00
3	c-type cytochrome biogenesis factor	14.4	0.77	0.65	0.75
4	Translation initiation factor	15.4	0.40	0.45	0.65
5	Ribosomal protein S9p	16.4	0.70	0.75	0.50
6	Polysulfide reductase subunit	21.0	0.00	0.00	0.00
7	Nucleoside diphosphate kinase	21.6	0.00	0.10	0.00
8	Tartrate dehydratase b-subunit	23.8	0.00	0.00	0.00
9	3-hexulose 6-phosphate synthase	23.1	0.65	0.60	0.65
10	Hydrogenase formation protein hypE	26.8	0.35	0.55	0.55
11	Methyltransferase	29.3	0.00	0.05	0.00
12	Chorismate mutase	29.3	0.70	0.70	0.65
13	Tyrosine t-RNA synthetase	36.0	0.95	0.95	0.95
14	nirD protein	36.7	0.70	0.45	0.25
15	Soluble hydrogenase	37.3	0.00	0.00	0.00
16	Aspartate-semialdehyde dehydrogenase	37.4	0.00	0.00	0.00
17	Phosphate cyclase	37.4	0.80	0.90	0.85
18	Purine-nucleoside phosphorylase	41.7	0.05	0.00	0.00

Protein = eighteen proteins from the hyperthermophilic archaeon *P. aerophilum* expressed in *E. coli* BL21(DE3) at 37°C from the pET T7 promoter. MW = theoretical molecular weight in kDa was calculated from amino acid sequence. Fraction soluble = fraction of total protein found in the soluble lysate as determined by SDS-PAGE densitometry for: non-fusion (NF); fused with C-terminal GFP S11 M3; or C-terminal ccGFP S11 v1. Relative uncertainty is $\pm$5%, average of three replicates.
